# O002: Patient and healthcare worker perception about patient participation in improving hand hygiene practices: impact of a patient participation intervention

**DOI:** 10.1186/2047-2994-2-S1-O2

**Published:** 2013-06-20

**Authors:** AJ Stewardson, N Farquet, A Gayet-Ageron, S Touveneau, Y Longtin, A Iten, D Pittet, H Sax

**Affiliations:** 1 The Univ. of Geneva Hosp. and Fac. of Medicine, Geneva, Switzerland; 2 Laval University, Quebec, Canada; 3 Univ. and University Hosp. of Zurich, Zurich, Switzerland

## Introduction

We implemented a cluster-randomized study at a 2200-bed academic medical centre to assess the impact of novel strategies to promote hand hygiene (HH). Wards in one of the three study arms were exposed to a patient participation (PP) program.

## Objectives

To investigate the impact of a formal PP program on healthcare worker (HCW) and patient perception of PP for improving HH compliance.

## Methods

We performed two cross-sectional studies with written, self-administered, anonymous questionnaires: one each for patients and HCWs. Adult patients were eligible if hospitalized for more than 24 hours in one or more of 66 study wards and discharged between May 16 and May 31, 2012 to their usual place of residence. Patients were defined as “exposed” (to PP) if they stayed ≥ 24 hours in ≥ one ward in the PP study arm during their admission. Completed surveys were returned via postal mail. HCWs working in all study wards were eligible. HCWs were defined as “exposed” if they currently worked in a PP ward. Surveys were brought to each study ward by a member of the study team and completed surveys were returned via internal mail. For each survey, non-respondents received reminders 2 and 4 weeks after initial distribution.

## Results

The response rate was similar among exposed and non-exposed patients: 167/316 (53%) and 378/686 (55%), respectively. Compared with non-exposed patients, exposed patients were no more likely to agree that “patients should remind healthcare workers to perform hand hygiene” (31% vs 26%, p=.25) or to report having reminded a nurse (5% vs 3%, p=.16) or a doctor (2% vs 5%, p=.29) during their last admission. The response rate was also similar among exposed and non-exposed HCWs: 230/531 (43%) and 436/999 (44%), respectively. The concept of patients reminding HCWs to perform HH was accepted by 67% of HCWs. HCW acceptance was independently associated with PP exposure (OR 1.51, CI95% 1.00-2.29, p=.048) and nursing profession (OR 1.69, CI95% 1.03-2.79, p=.039).

## Conclusion

Compared with control wards, HCWs (but not patients) present in intervention wards had a more favourable perception of PP in HH promotion.

## Disclosure of interest

None declared.

